# Treatment of Esophageal Hematoma After Left Atrial Appendage Occlusion: A Case Report

**DOI:** 10.3389/fcvm.2022.941924

**Published:** 2022-06-23

**Authors:** Chaodi Cheng, Yang Zhou, Yanjiang Wang, Liang Shi, Ying Tian, Xingpeng Liu

**Affiliations:** Heart Center, Beijing Chaoyang Hospital, Capital Medical University, Beijing, China

**Keywords:** left atrial appendage occlusion, transesophageal echocardiography, esophageal hematoma, atrial fibrillation, complication

## Abstract

Left atrial appendage occlusion (LAAO) is an alternative to oral anticoagulation therapy in patients with atrial fibrillation who are at high risk for bleeding and thromboembolic events. Transesophageal echocardiography (TEE) is the standard modality for intraprocedural imaging during LAAO. We report a rare case of extensive submucosal esophageal hematoma that developed after a TEE-guided LAAO procedure. The cause, management, and prevention of this complication are explored in depth in this report.

## Introduction

Anticoagulation is recommended for most patients with atrial fibrillation (AF) because this population is at high risk for stroke and other thromboembolic events. However, long-term anticoagulation therapy is contraindicated in certain cases. As reported in previous studies, the left atrial appendage (LAA) is the primary origin site of 90% of thrombi in patients with AF (1). Because the LAA morphology creates a favorable environment for blood stasis, the occlusion, ligation, and amputation of the LAA are reasonable alternatives to anticoagulation. These interventions are especially helpful for offsetting the high risk for bleeding events in patients with non-valvular AF who do not tolerate long-term anticoagulation. Transesophageal echocardiography (TEE) is widely used for intraprocedural imaging during left atrial appendage occlusion (LAAO) because it is a cost-effective modality that enables good visualization of the LAA.

## Case Description

A 70-year-old man with persistent AF and a history of thrombus formation in the LAA was referred to our hospital for LAAO. The patient had previously undergone left atrial anterior wall linear ablation with electrical isolation of the LAA. At admission, the patient had high CHA2DS2-VASc and HAS-BLED scores (5 and 3, respectively). The preprocedural TEE ruled out new thrombus formation in the LAA.

**FIGURE 1:**
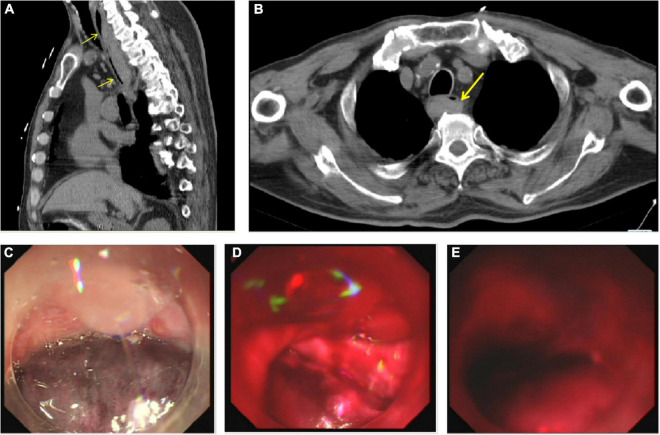
**(A)** Sagittal chest computed tomography (CT) image reconstruction showed the hematoma extending from the entrance of the esophagus to the gastroesophageal junction (yellow arrows). **(B)** Axial chest CT image reconstruction showed the hematoma occupying more than half of the lumen (yellow arrow). **(C)** Endoscopy revealed esophageal mucosal exfoliation in the upper third of the esophagus, **(D)** middle third of the esophagus, and **(E)** lower third of the esophagus.

**FIGURE 2:**
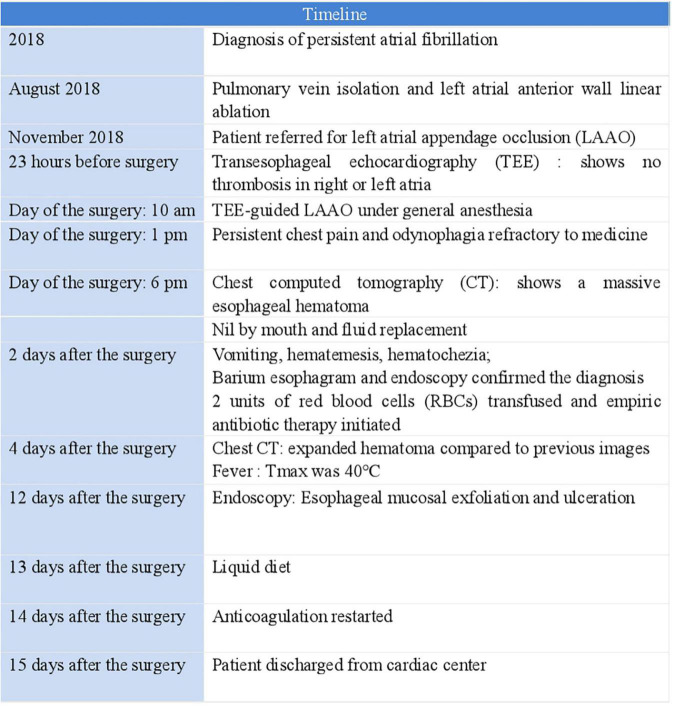
Timeline.

After TEE-guided transseptal puncture (TSP), an intravenous bolus of heparin (10,000 IUs) was administered to achieve a target activated clotting time (ACT) of 250–300 s. A 27-mm WATCHMAN device was then successfully implanted into the LAA. Twenty minutes after the patient returned to the ward, he reported excruciating substernal chest pain and odynophagia that was persistent and refractory to medical therapy. An emergency computed tomography (CT) scan of the chest revealed a massive esophageal hematoma that extended from the entrance of the esophagus down to the gastroesophageal junction, occupying more than half of the lumen (Figures 1A,B); atrio-esophageal fistula was ruled out based on the chest CT scan and barium esophagram. Endoscopy later confirmed the diagnosis of esophageal hematoma and demonstrated esophageal mucosal exfoliation (Figures 1C–E). Anticoagulation therapy was discontinued immediately. The patient’s timeline is shown in Figure 2.

In the following days, the patient experienced vomiting, hematemesis, hematochezia, and a dramatic decrease in the hemoglobin level. After discussion with a multidisciplinary team, we adopted conservative management with nil by mouth orders and intravenous fluid replacement. Cefmetazole was administered as the Empiric perioperative prophylactic antibiotic. Four days after the procedure, the patient became febrile; the highest temperature recorded was 40°C. A routine blood test showed a normal leukocyte count of 6.57 × 10^9^/L (normal range, 3.5–9.5 × 10^9^/L), a normal platelet count of 137 × 10^9^/L (normal range, 100–300 × 10^9^/L), and a mildly decreased hemoglobin level of 124 g/L (normal range, 130–175 g/L). After multiple negative blood cultures, we concluded that the patient experienced absorption fever. Nevertheless, the antibiotic therapy was upgraded to piperacillin-tazobactam because we could not exclude a rupture of the esophageal hematoma and the high risk for secondary esophageal perforation. The broad-spectrum antimicrobial therapy was continued until the fever eventually subsided. After gradual symptomatic relief and a steady rise in the hemoglobin level, warfarin anticoagulation therapy was reintroduced under close monitoring. Fortunately, neither device-related thrombus (DRT) nor ischemic stroke occurred within this time window. The patient was asymptomatic at discharge from the hospital, and the follow-up endoscopy showed complete resolution of the hematoma.

## Discussion

Esophageal hematoma is an uncommon clinical condition that may present with gradual onset of chest pain, odynophagia, dysphagia, hematemesis, and melena. The pathogenesis of the submucosal esophageal hematoma has not been well documented, but can likely be attributed to a dissection of the submucosal muscle layers resulting from a submucosal hemorrhage (2). The causes of non-iatrogenic esophageal hematoma include foreign body ingestion, esophageal malignancy, trauma, or other esophageal pathologies such as esophagitis. According to previous case reports, iatrogenic esophageal hematoma may occur after AF ablation procedures (3), upper gastrointestinal endoscopy, or thrombolytic therapy for pulmonary embolism that leads to abnormalities in hemostasis (4). Although it typically has a good prognosis, with resolution of the symptoms within weeks, esophageal hematoma can result in nearly complete occlusion of the gastric lumen, or even compression of the adjacent cardiac chambers and airway, causing dyspnea or pulmonary atelectasis. An initially small mucosal tear may expand and subsequently evolve to rupture and fatal GI bleeding. Conservative therapies such as analgesics, proton pump inhibitors, bowel rest, and discontinuation of anticoagulation therapy remain the mainstay of management in most cases. Urgent surgical intervention is necessary when hemodynamic collapse occurs. The differential diagnosis includes myocardial infarction, pulmonary embolism, atrio-esophageal fistula, and esophageal perforation.

In this case report, we describe the evolution and management of a submucosal esophageal hematoma that developed after a TEE-guided LAAO procedure. Some risk factors may increase the odds of developing this rare complication. Manipulation of the TEE probe is the first and foremost of these risk factors. In this case, TEE was performed both before and during the LAAO procedure. When a patient undergoes TEE twice within 24 h, the esophagus becomes vulnerable to direct mechanical trauma, which may result in esophageal injuries after structural cardiac intervention such as LAAO. Furthermore, TEE requires general anesthesia; in this setting, the patient cannot swallow to facilitate probe insertion or react to pain, which may mask the diagnosis and delay treatment. Postoperative nausea and vomiting associated with general anesthesia may further increase the risk of esophageal injury in such cases. Freitas-Ferraz et al. reported that 86% of patients have a new injury after TEE-guided intervention, with 40% experiencing complex injuries such as intramural hematoma and mucosal laceration (5). Notably, advanced age, prolonged procedural time, and previous esophageal or gastric pathology may also contribute to the TEE-related complications. To prevent TEE-related esophageal hematoma after LAAO, caution is recommended when selecting patients to undergo TEE-assisted procedures. For instance, TTE should be avoided in patients with known esophageal disease.

Another underlying mechanism that predisposes patients to the development of submucosal hemorrhage is the abnormal hemostasis induced by anticoagulation. In recent years, research has provided increasing evidence about the safety and efficacy of anticoagulation therapy after LAAO procedures. However, to our knowledge, there is a limited number of studies about the effects of anticoagulation during LAAO procedures, particularly with regard to the association between heparin dosage and major bleeding complications. In the PROTECT AF study, heparin was administered to maintain an ACT > 200 s (6). In the updated consensus statement, the authors recommend administering unfractionated heparin (70–100 IU/kg) during the implantation procedure, before or immediately after the TSP, to maintain an ACT ≥ 250 s (7). After implantation of the WATCHMAN device, warfarin anticoagulation (INR 2–3) should be initiated and continued for 45 days, followed by administration of clopidogrel for 6 months in patients with a low bleeding risk. In this case, bridging therapy with low-molecular-weight heparin (dalteparin 100 IU/kg q12 h) was initiated 4 days before the procedure, halted when the complication was recognized, and restarted on day 14 after the procedure, once the symptoms of esophageal hematoma resolved. The patient was switched to warfarin anticoagulation on day 19 after the procedure. Although we did not perform a control TEE, there were no clinical arguments for embolism formation in the atria or device-related thrombus during the hospitalization and follow-up. More evidence is needed to determine the optimal anticoagulation protocols during LAAO and specific heparin dosing adjustments for individuals with a high risk for bleeding. Additionally, to balance the risks of thrombus formation and bleeding, a multidisciplinary team should be involved in the decisions regarding the timing of anticoagulation therapy.

Cardiac computed tomography angiography (CCTA) is a sensible alternative modality to TEE before procedures. Despite the radiation exposure and equipment requirements associated with CCTA, this imaging modality offers a higher spatial resolution than TEE, demonstrating non-inferiority or advantages over TEE in terms of thrombus detection, characterization of the LAA anatomy, and LAA measurement (8). Intracardiac echocardiography (ICE) is another potential alternative to TEE for imaging during LAAO procedures. ICE has a higher cost and longer procedural time compared with TEE. However, ICE-guided operations require only local anesthesia or conscious sedation, are safe, and their success rates and incidence of adverse events are comparable to those of TEE-guided procedures (9). In addition, novel techniques or imaging systems such as echocardiographic-fluoroscopic fusion imaging and 3D TEE, although not yet widely accepted, may offer certain advantages when it comes to guiding LAAO procedures (10).

In conclusion, esophageal hematoma is a rare complication of TEE-guided LAAO procedures. Abnormal hemostasis, inadequate manipulation of the TEE probe, and previous esophageal diseases can increase the risk for developing this complication. Additional evidence is needed to determine the adequate protocols for heparin dosing and monitoring during LAAO procedures, with the aim of preventing this complication. Meanwhile, caution is recommended when evaluating and selecting patients for TEE-guided intervention. If possible, clinicians should avoid performing repeat TEE on the same patient in a short period of time. It is also important to note that a multidisciplinary approach is essential for devising the most effective strategies tailored to emergent cases.

## Data Availability Statement

The raw data supporting the conclusions of this article will be made available by the authors, without undue reservation.

## Ethics Statement

The studies involving human participants were reviewed and approved by the Human Research Committee of Beijing Chaoyang Hospital. The patients/participants provided their written informed consent to participate in this case study and for the publication of any potentially identifiable images or data included in this article.

## Author Contributions

CC and YZ drafted the manuscript and were responsible for the collection of data and analysis. XL revised the manuscript. All authors read and approved the final manuscript.

## Conflict of Interest

The authors declare that the research was conducted in the absence of any commercial or financial relationships that could be construed as a potential conflict of interest.

## Publisher’s Note

All claims expressed in this article are solely those of the authors and do not necessarily represent those of their affiliated organizations, or those of the publisher, the editors and the reviewers. Any product that may be evaluated in this article, or claim that may be made by its manufacturer, is not guaranteed or endorsed by the publisher.
